# From *Fusarium solani* Keratitis to Full Visual Acuity: A Case of Infection Requiring Multiple Corneal Transplants

**DOI:** 10.3390/jof12080619

**Published:** 2026-08-18

**Authors:** Franziska Harzer, Adrian Gericke, Joanna Wasielica-Poslednik

**Affiliations:** Department of Ophthalmology, University Medical Center of the Johannes Gutenberg University Mainz, Langenbeckstr. 1, 55131 Mainz, Germanyjoanna.wasielica-poslednik@unimedizin-mainz.de (J.W.-P.)

**Keywords:** keratitis, *Fusarium solani*, visual outcome, penetrating keratoplasty, DMEK

## Abstract

Background and Clinical Significance: The *Fusarium solani* species complex is a group of filamentous fungi capable of causing severe ocular infections. It is a frequent cause of mycotic keratitis, which is associated with a poor prognosis due to aggressive biological behavior and resistance to most antifungal agents. Contact lens wear is a well-known risk factor; Case Presentation: A 48-year-old female contact lens wearer presented with a persistent corneal ulcer and hypopyon in the right eye. Initial visual acuity was hand movements. Hyphae of the *Fusarium solani* species complex were detected in an anterior chamber punctate sample. Intensive topical and systemic antifungal therapy was initiated, followed by therapeutic penetrating keratoplasty (PK) without corticosteroid treatment. Two months later, a second optical PK was performed. Over the subsequent two years, the patient underwent cataract surgery and developed endothelial corneal decompensation. During long-term follow-up, three Descemet membrane endothelial keratoplasties (DMEK) were required due to recurrent endothelial failures. Seven years after the initial presentation, the eye remained clinically stable with a best-corrected visual acuity of 0.0 logMAR; Conclusions: Early therapeutic PK without corticosteroid treatment was likely a key factor in successful infection control in this case of *Fusarium solani* keratitis. Full visual recovery can be achieved even after multiple penetrating and lamellar corneal transplantations in severe fungal keratitis.

## 1. Introduction and Clinical Significance

The *Fusarium solani* complex is a species complex within the group of filamentous *Fusarium* fungi. Members of this genus are ubiquitous in the environment, for example in soil, on plants, or in water, and are mostly harmless to humans [1]. However, certain species within this group, particularly those belonging to the *Fusarium solani* complex, can cause severe infections. This complex is frequently associated with mycotic keratitis, a leading cause of monocular blindness in tropical and subtropical regions. In Western countries, women are predominantly affected by *Fusarium* keratitis, with contact lens use being a significant risk factor [2,3]. The treatment of this disease is a major challenge, as *Fusarium* species are resistant to many antifungal drugs. The outcome is often poor. In a German study, nine out of thirteen eyes with confirmed *Fusarium* infection underwent keratoplasty and three eyes had to be enucleated [3]. Rapid diagnosis and prompt, appropriate treatment are essential to preserve the affected eye and maintain visual acuity [4]. A prolonged interval between symptom onset and diagnosis is a known risk factor for unfavorable outcomes [3]. Therapeutic penetrating keratoplasty (PK) is a well-established treatment option for severe corneal infections [5]. Studies on postoperative management of fungal keratitis indicate that topical corticosteroids should generally be avoided in the early period following therapeutic PK, due to concerns about promoting fungal recurrence and exacerbating infection. Alternative strategies include withholding corticosteroids, using other forms of immunosuppressive therapy, or initiating steroid treatment after a delay—typically one to two weeks postoperatively and only in the absence of clinical signs of recurrence. These approaches have been reported to control intraocular inflammation without significantly increasing the risk of fungal recurrence [6].

Despite prolonged symptoms prior to diagnosis, advanced findings at initial presentation, and the need for extended treatment involving multiple corneal transplants, this case demonstrates a highly favorable outcome following keratitis caused by *Fusarium solani*. We aim to report on our experience with the combined surgical and medical management employed in this case.

## 2. Case Presentation

### 2.1. Medical History and Clinical Findings at First Presentation

**Figure 1:**
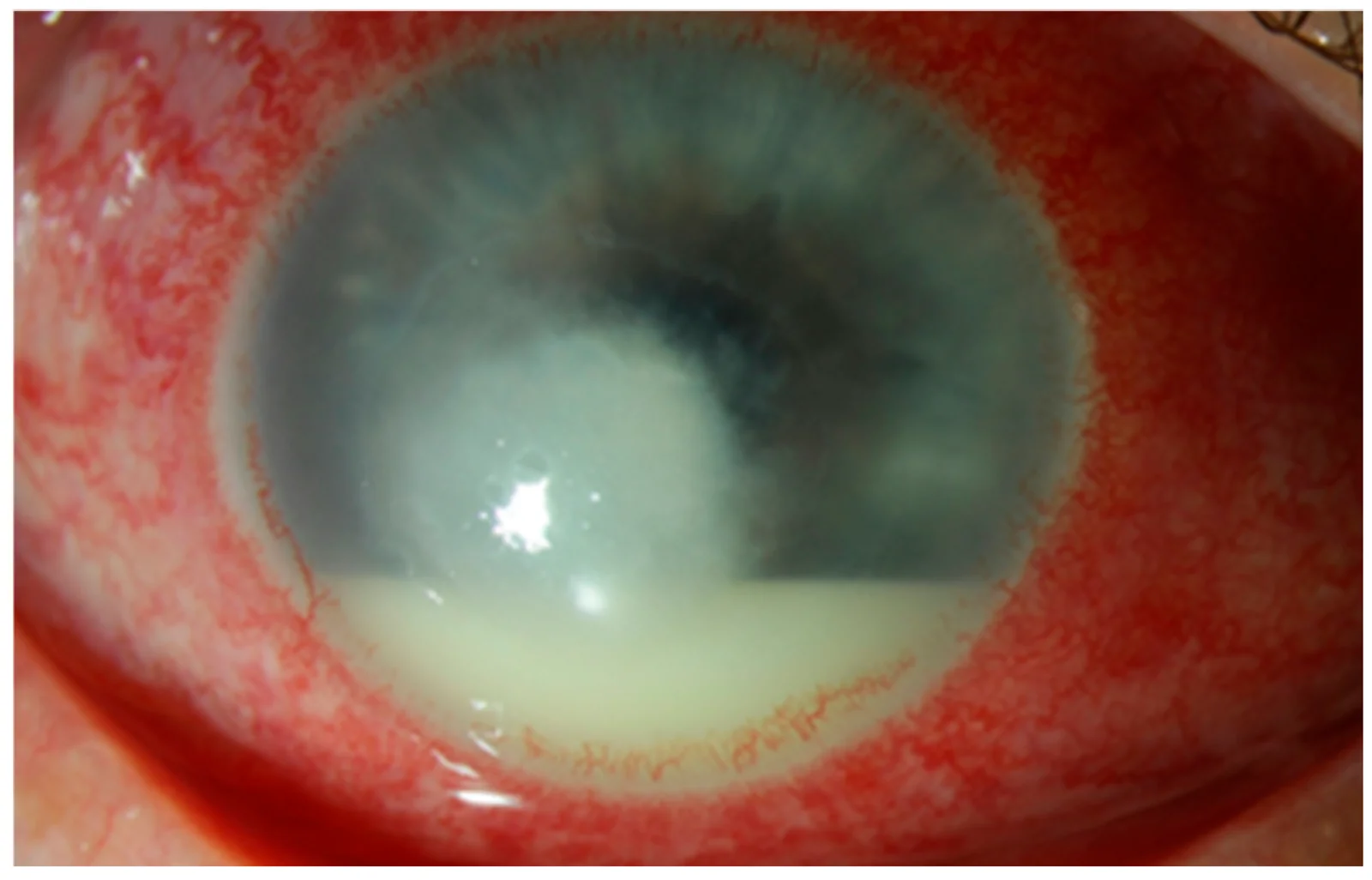
Corneal ulcer and hypopyon in the right eye at initial presentation.

A 48-year-old Caucasian woman presented with a persistent corneal ulcer and hypopyon in the right eye that was unresponsive to prior inpatient treatment with topical ofloxacin and intravenous ciprofloxacin. Slit-lamp examination revealed a 5 mm central corneal ulcer with whitish infiltrates and a 3 mm hypopyon (Figure 1). The patient was a monthly soft contact lens wearer with high myopia in both eyes. At presentation, corrected distance visual acuity was limited to hand movements in the right eye and 0.3 logMAR in the left eye.

### 2.2. Initial Treatment

The patient received systemic therapy with cefuroxime 500 mg twice daily, linezolid 600 mg twice daily, and voriconazole 400 mg twice daily. Initial topical treatment with moxifloxacin 0.5% and a combination of polymyxin B, neomycin, and gramicidin was changed after three days to gentamicin, voriconazole 2%, and ciclosporin 2%. Natamycin 5% eye drops were subsequently added. Following anterior chamber irrigation with intracameral cefotaxime and voriconazole, microbiological examination revealed hyphae of the *Fusarium solani* species complex in the anterior chamber aspirate and on the contact lens surface. Antimicrobial susceptibility testing performed on the fungal culture demonstrated sensitivity to clindamycin, vancomycin, and linezolid, as well as amphotericin B, natamycin, and voriconazole. For amphotericin B, the minimum inhibitory concentration (MIC value) was 1 µg/mL; for natamycin and voriconazole, it was 4 µg/mL. The mold showed resistance to all other tested azoles, as well as caspofungine, with MIC values being higher than 8 µg/mL.

### 2.3. Diagnosis

Right Eye: *Fusarium solani* complex-associated fungal keratitis with hypopyon related to monthly soft contact lens wear.

### 2.4. Further Treatment and Course

One week later, due to recurrent hypopyon, a therapeutic penetrating keratoplasty (PK) with posterior synechiolysis was performed. Postoperatively, topical corticosteroids were deliberately withheld. At discharge, the corneal graft was clear, and visual acuity was 1.5 logMAR. As the ultrasound showed no signs of intraocular inflammation, systemic therapy was reduced to voriconazole 200 mg twice daily while topical treatment with natamycin 5%, voriconazole 2%, gentamicin, and ciclosporin 2% eye drops was maintained.

**Figure 2:**
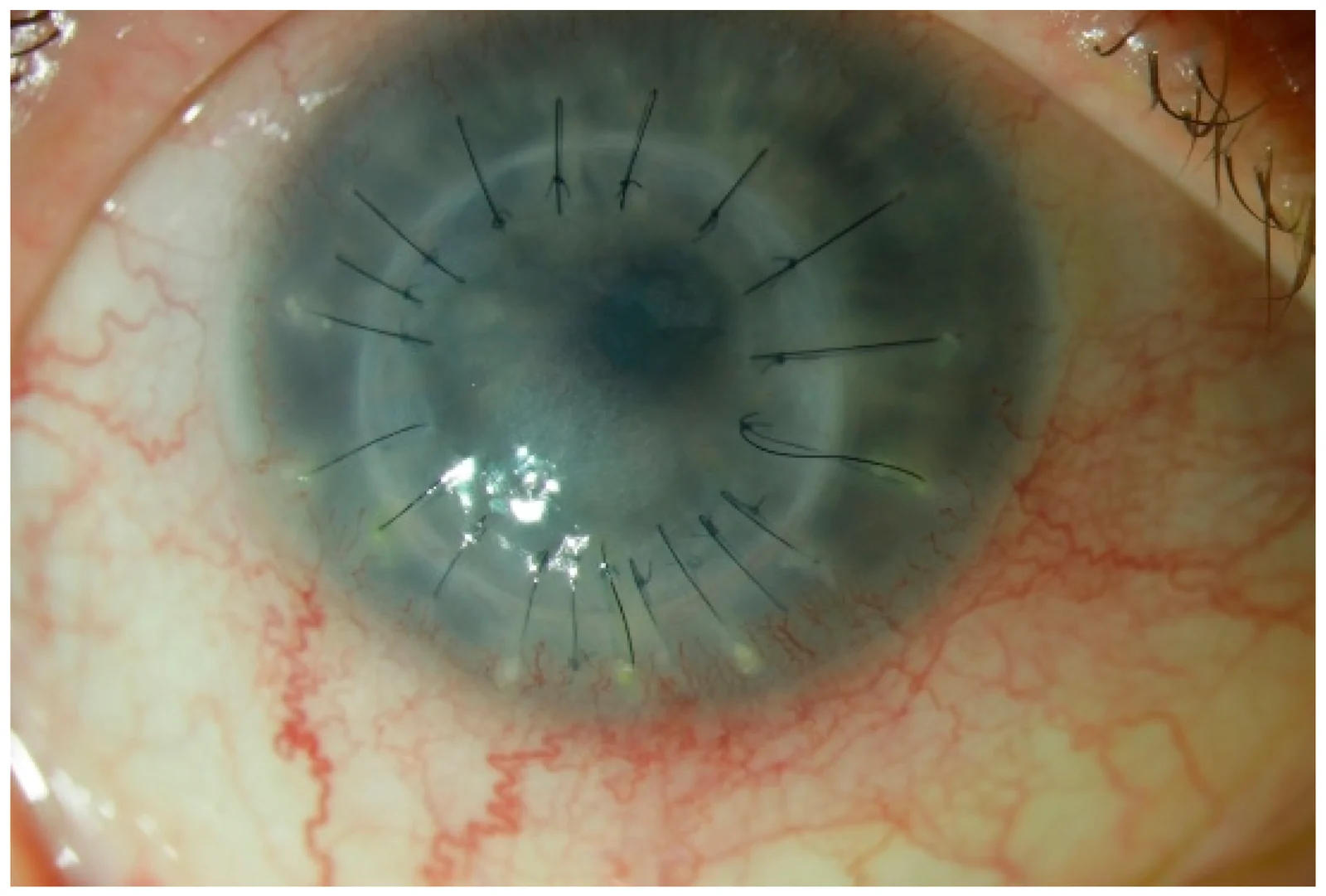
Immune graft rejection after therapeutic penetrating keratoplasty in the absence of corticosteroid treatment.

Within the following month, signs of expected immune graft rejection developed, including epithelial edema and loose sutures, but without evidence of recurrent fungal infection (Figure 2). Visual acuity in the right eye was reduced to counting fingers. A second PK was performed two months after the initial transplant. Topical therapy was subsequently switched to dexamethasone eye drops 6×/day, with a gradual taper of one drop per month to a maintenance dose of 1×/day, along with voriconazole 2% eye drops 2×/day. As the pathology report showed no evidence of fungal infection, other topical antifungals as well as systemic voriconazole were discontinued. Systemic immunosuppression with mycophenolate mofetil 1000 mg twice daily was administered for three months. Additionally, oral prednisone 30 mg once daily was initiated and tapered over 12 days. Due to a steroid-induced increase in intraocular pressure (IOP), dexamethasone eye drops were reduced to 2×/day, ciclosporine 1% eye drops were started twice daily, and topical antiglaucomatous therapy was initiated with clonidine, dorzolamide/timolol, and latanoprost. Voriconazole 2% eye drops were discontinued. The eye subsequently stabilized, and three months later visual acuity improved to 0.5 logMAR. Clinical examination revealed a clear graft, a synechia at the 6 o’clock position, and a cortical cataract. IOP stabilized between 10 and 15 mmHg, allowing discontinuation of clonidine eye drops.

**Figure 3:**
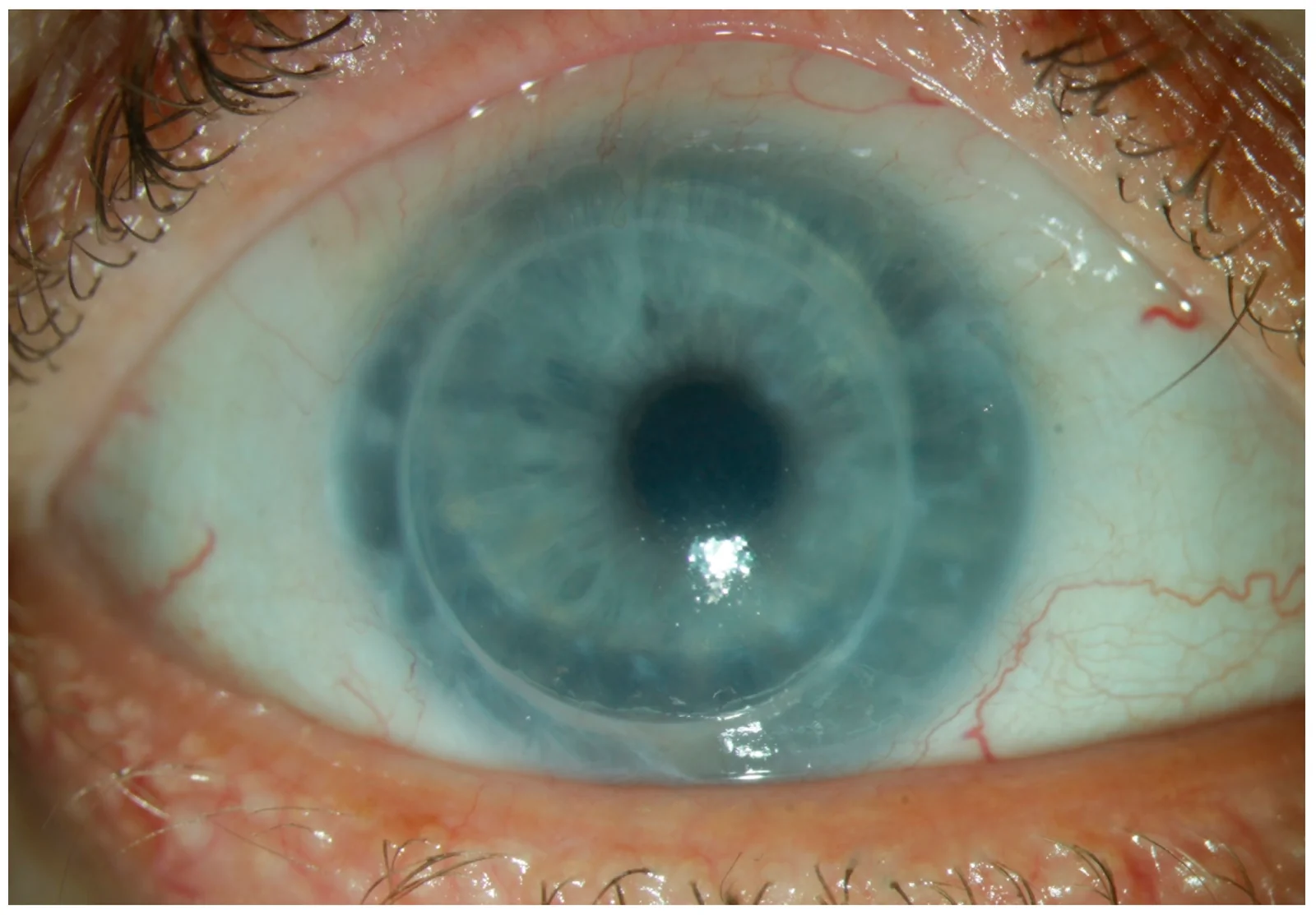
Endothelial corneal decompensation with anterior chamber irritation following cataract surgery.

Eighteen months after the second PK and five months after suture removal, cataract surgery with implantation of a toric intraocular lens was performed on the right eye. Four months later, the patient presented again with corneal edema, anterior chamber inflammation, and endothelial precipitates (Figure 3).

**Figure 4:**
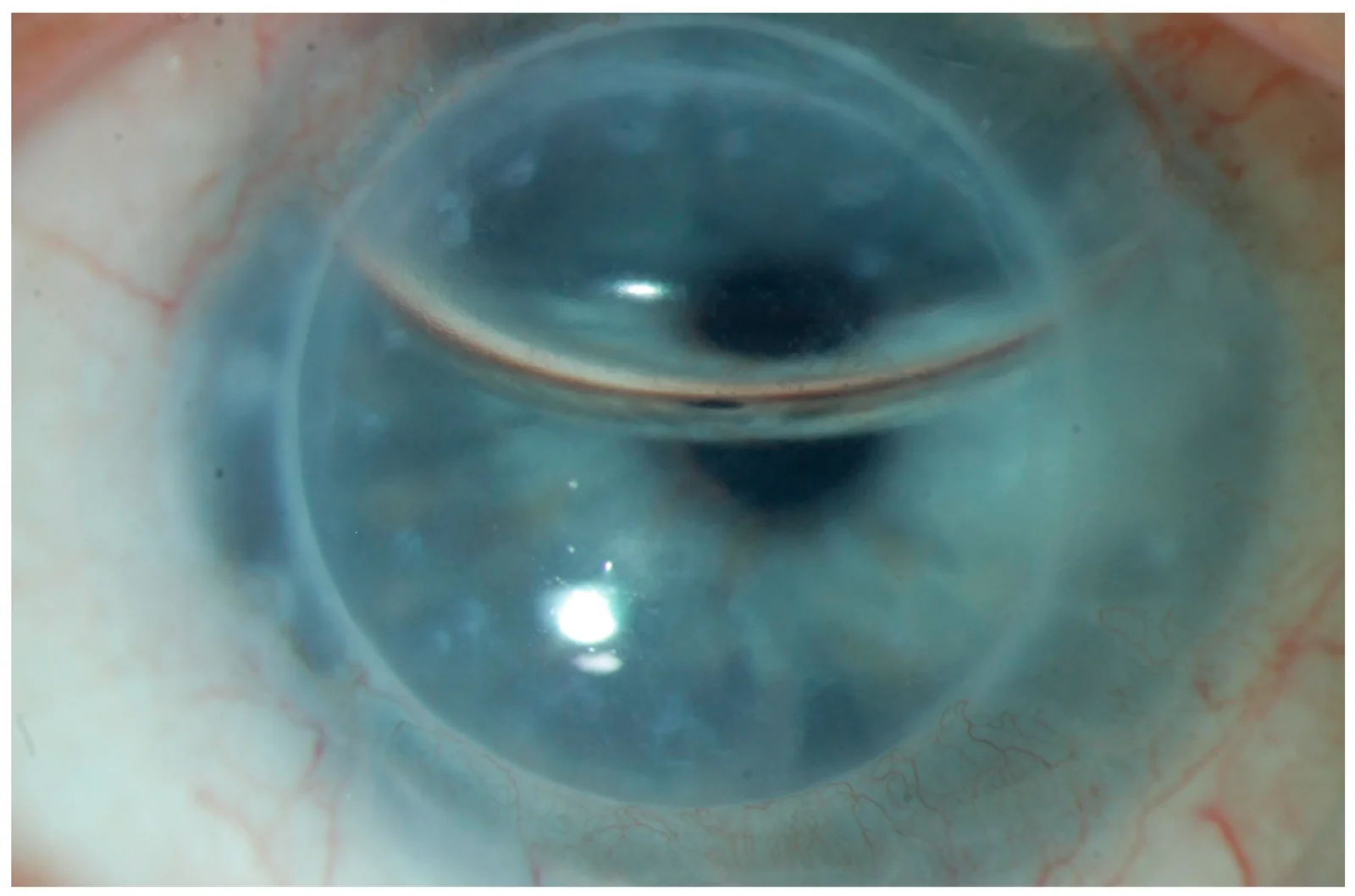
Eye after third DMEK.

Due to suspicion of herpetic keratouveitis, treatment with topical moxifloxacin, ganciclovir eye gel, and prednisolone eye drops, along with intravenous acyclovir, was initiated. An anterior chamber puncture, followed by PCR exam, revealed no evidence of CMV, HSV, or VZV, allowing discontinuation of antiviral therapy. Because of persistent endothelial corneal decompensation, Descemet membrane endothelial keratoplasty (DMEK) was performed without complications. Two weeks later, graft dehiscence required re-bubbling, and an IOP rise up to 30 mmHg occurred, necessitating topical IOP-lowering therapy. More than two months after surgery, epithelial edema persisted; therefore, a second DMEK was performed without complications. Due to nasal graft dehiscence detected two weeks after the second DMEK, another re-bubbling was required. Visual acuity in the right eye subsequently improved to 0.4 logMAR four months later. Six months after the second DMEK, recurrent graft failure with epithelial edema occurred, and a third DMEK was performed (Figure 4). One year later, the clinical findings remained stable, and visual acuity improved to 0.1 logMAR. In the meantime, the right eye has undergone Nd:YAG laser capsulotomy.

### 2.5. Follow-Up and Outcome

**Figure 5:**
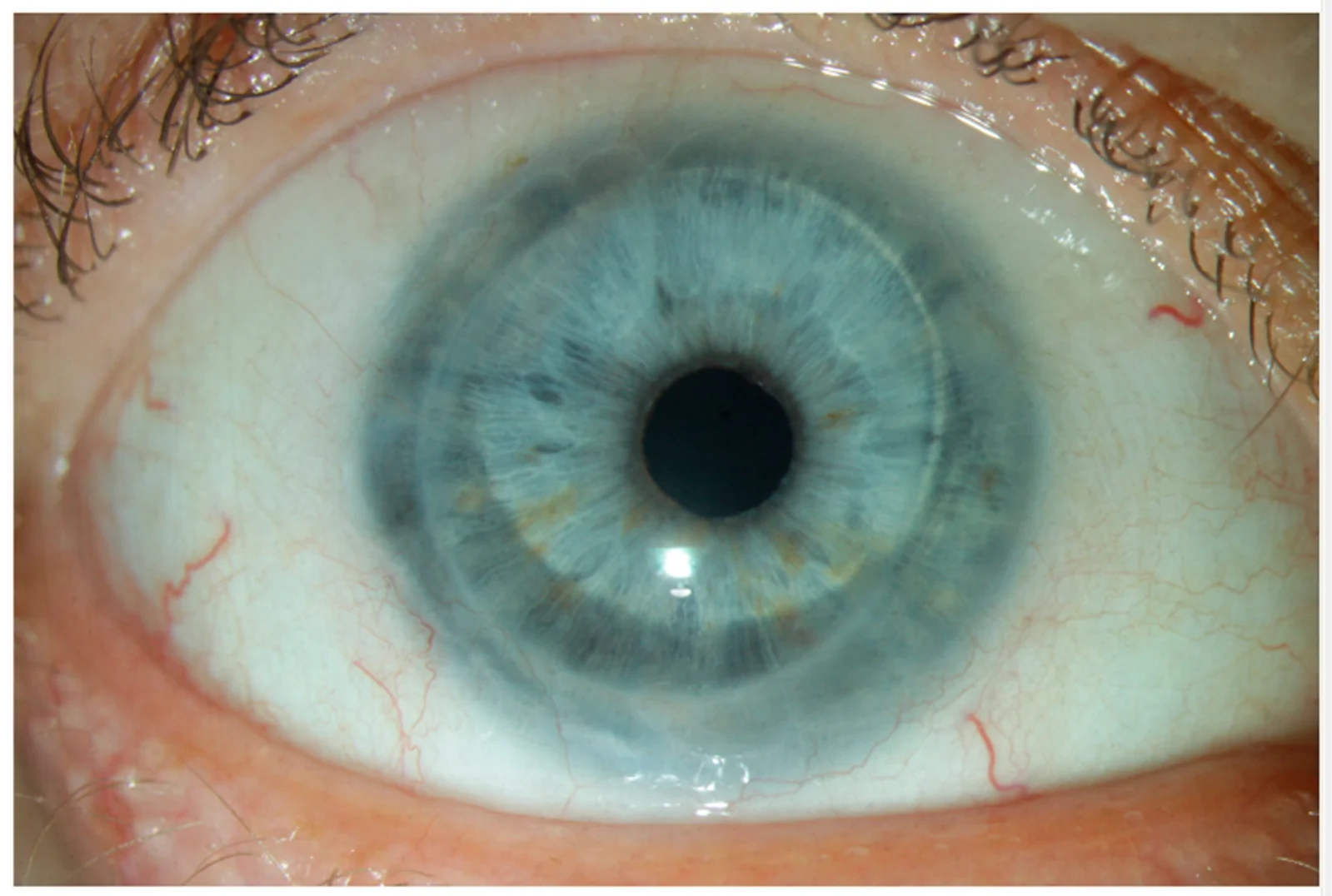
Eye at the patient’s last appointment, seven years after the initial presentation.

Seven years have passed since the initial presentation with fungal keratitis. At the patient’s last follow-up examination, corrected distance visual acuity in the right eye was 0.0 logMAR. The corneal graft was clear (Figure 5). Topical dexamethasone eye drops once daily and artificial tears are being continued.

Table 1 provides a chronological overview of the interventions performed on the right eye over time.

## 3. Discussion

While keratitis caused by *Fusarium solani* was long considered a disease predominantly occurring in tropical regions, an increasing number of cases has been reported in Europe over the last 20 years. The main risk factor for *Fusarium* keratitis in developed countries is the use of soft contact lenses [7]. Other risk factors include low socioeconomic status, immunosuppression, diabetes, HIV infection, as well as chronic topical steroid use [6,7].

When *Fusarium* keratitis is suspected, early initiation of local antifungal therapy, including voriconazole 2% and natamycin 5%, is essential [7]. Systemic voriconazole can be added in advanced cases. Some authors recommend performing penetrating keratoplasty within one week if the response to local antifungal medication is unsatisfactory. Steroids are contraindicated both pre- and postoperatively [7]. In general, the visual prognosis is poor [3]. A significant risk factor for an unfavorable visual outcome is a long interval between the onset of symptoms and diagnosis [7]. Furthermore, a study from India including 97 patients with keratitis caused by *Fusarium solani* found that central ulcers, ulcer size > 4 mm^2^, and the need for surgical intervention were significantly associated with poorer visual outcomes. In that study, visual acuity at presentation was also associated with the final best corrected visual acuity [8]. In our case, not only were multiple surgeries required, but the initial ulcer size was also nearly 20 mm^2^.

Topical treatment with antifungals is often difficult, as antifungal drugs frequently cannot reach pathogens located deep within the cornea. Therefore, with some medications, regular corneal abrasion or repeated intracameral or intrastromal administration is required. In addition, primary resistance is common [9]. Several antifungal agents are used in the treatment of *Fusarium* keratitis. Azoles such as voriconazole have good corneal penetration, meaning that corneal abrasion is usually not necessary. Voriconazole can also be administered intravenously, intracamerally or intrastromally [9]. Polyenes such as amphotericin B and natamycin have a molecular weight that is too high to allow adequate corneal penetration [10]. In advanced cases, combination therapy is recommended. Topical natamycin 5% or amphotericin B 0.5% can be combined with voriconazole 2% and applied alternately every 30 min [7,10]. In particular, natamycin 5% eye drops have demonstrated effectiveness in cases of *Fusarium* keratitis [7,9]. Yet, these eye drops are not easily available in many developed countries such as Germany [7].

In our case, the correct diagnosis was established relatively quickly due to an early anterior chamber puncture, in which the *Fusarium solani* species was detected. Fortunately, the antibiogram showed no resistance to the therapy that had already been initiated, so the treatment could be continued.

After therapeutic PK, the conscious decision was made to refrain from administering postoperative steroids. This approach is consistent with the current literature. Szalinski et al. and Kovalchuk et al. recommend waiting at least 10–14 days after keratoplasty before slowly initiating steroids, provided that no clinical signs of recurrence are present [7,11]. While steroids can prevent graft rejection, there is always a risk of aggravation or recurrence of the fungal infection [6].

In our case, we decided to completely avoid steroids after the first PK. This meant that rejection after the first surgery was likely inevitable; however, we consciously accepted this risk. Fortunately, this approach worked out well, and no recurrence of the fungal infection occurred in our patient’s eye.

After the second PK, immunosuppression according to our clinical routine was initiated to prevent rejection. At the same time, as there was no evidence of recurring fungal infection, we discontinued systemic antifungal therapy. Due to a subsequent steroid-induced increase in intraocular pressure, dexamethasone eye drops were reduced, ciclosporin eye drops were added, and antiglaucomatous therapy was initiated.

There are three possible differential diagnoses for endothelial decompensation after cataract surgery: endothelial immune rejection, viral infection, and endothelial failure. Since neither corticosteroid therapy nor anti-herpetic therapy led to improvement in the patient’s condition, and anterior chamber puncture did not reveal any pathogens, endothelial decompensation was assumed. Therefore, a DMEK was performed. A repeat PK was ruled out because of its greater invasiveness, a higher risk of rejection [12] and the toric intraocular lens that had already been implanted.

Re-bubbling following DMEK after penetrating keratoplasty is much more common than after primary DMEK and ranges from 6.3% to 100% [13], whereas the re-bubbling rate after primary DMEK ranges from 0% to 44% [14]. Additionally, graft detachment in DMEK after PK appears to occur later but tends to be more extensive compared with primary DMEK [13]. In our case, graft failure may also have been associated with the increase in intraocular pressure. DMEK can be repeated, as was done in our case.

Despite the prolonged symptoms before diagnosis, the advanced findings at initial presentation, and the lengthy treatment involving multiple corneal transplantations, the present case demonstrates a very favorable outcome following keratitis caused by *Fusarium solani*. However, such cases require considerable patience from both the treating physician and the patient. Patients should be informed about the prolonged duration of treatment and, when necessary, provided with psychological support.

## 4. Conclusions

This case report shows that—even though it might be a rare outcome in eyes severely affected by *Fusarium solani* keratitis—full visual acuity can be achieved with local and systemic therapy, regular monitoring, multiple surgeries and great perseverance on the part of the patient as well as the treating physicians.

## Figures and Tables

**Table 1:** Interventions in the course of time. All surgeries were performed on the right eye.

Course of Time	Type of Surgery	Systemic Therapy
Year 1, June	Anterior chamber punctate	Antifungal
Year 1, July	Therapeutic PK + posterior synechiolysis	
Year 1, September	Second PK	Steroids
Year 1, December	Synechiolysis	
Year 3, February	Cataract surgery	
Year 3, June	Anterior chamber puncture	Acyclovir
Year 3, November	DMEK, Re-Bubbling	
Year 4, March	Second DMEK, Re-Bubbling	
Year 5, February	Third DMEK	
Year 5, October	Nd:YAG Laser Capsulotomy	

## Data Availability

The original contributions presented in this study are included in the article. Further inquiries can be directed to the corresponding author.

## Abbreviations

The following abbreviations are used in this manuscript:

CMV	Cytomegalovirus
DMEK	Descemet Membrane Endothelial Keratoplasty
HIV	Human Imuunodeficiency Virus
HSV	Herpes Simplex Virus
IOP	Intraocular Pressure
MIC	Minimum inhibitory concentration
PK	Penetrating Keratoplasty
VZV	Varicella Zoster Virus
